# Ocular biometry with swept-source optical coherence tomography-based optical biometer in Japanese patients with *EYS*-related retinitis pigmentosa: a retrospective study

**DOI:** 10.1186/s12886-022-02284-3

**Published:** 2022-02-02

**Authors:** Daiki Sakai, Satoshi Yokota, Akiko Maeda, Yasuhiko Hirami, Makoto Nakamura, Yasuo Kurimoto

**Affiliations:** 1 Department of Ophthalmology, Kobe City Eye Hospital, Kobe, Japan; 2 Department of Ophthalmology, Kobe City Medical Center General Hospital, Kobe, Japan; 3 Department of Surgery, Division of Ophthalmology, Kobe University Graduate School of Medicine, Kobe, Japan

**Keywords:** Retinitis pigmentosa, Ocular biometry, Causative genes,, EYS, Astigmatism, Keratometry

## Abstract

**Background:**

This study aimed to identify the features of ocular biometry in patients with *EYS*-related retinitis pigmentosa using IOLMaster 700.

**Methods:**

We retrospectively reviewed the medical records of patients with retinitis pigmentosa. Patients with records of the following were included: (1) ocular biometry measurements using the IOLMaster 700 and (2) genetic diagnostic tests. Axial length, keratometry, anterior chamber depth, aqueous depth, lens thickness, central corneal thickness (CCT), and corneal diameter (white to white) measurements were extracted. Based on keratometry measurements, (1) standard keratometric astigmatism, (2) posterior corneal astigmatism, and (3) total corneal astigmatism were obtained. Demographics and biometric parameters were compared between patients with *EYS*-related retinitis pigmentosa and other patients with retinitis pigmentosa.

**Results:**

A total of 86 eyes of 44 patients (23 females and 21 males; mean age: 47.7 years) with retinitis pigmentosa were included. Of these, 18 were identified as having *EYS* variants. CCT was significantly thinner (*P* < 0.001) and the posterior corneal curvature at the steepest meridian was significantly smaller (*P* = 0.024) in patients with *EYS*-related retinitis pigmentosa than in other patients with retinitis pigmentosa. The magnitudes of all corneal astigmatism measurements was higher in patients with *EYS*-related RP, although these differences were not statistically significant.

**Conclusion:**

Patients with *EYS*-related retinitis pigmentosa had unique features in ocular biometry, such as thinner central corneal thickness and smaller posterior corneal curvature radius at the steepest meridian compared with other patients with retinitis pigmentosa. The findings suggest that patients with retinitis pigmentosa have different ocular dimension features among the different causative genes.

**Supplementary Information:**

The online version contains supplementary material available at 10.1186/s12886-022-02284-3.

## Background

Retinitis pigmentosa (RP) is the most common hereditary retinal dystrophy, with a worldwide prevalence of approximately 1 in 4000 individuals [1]. The clinical course of RP is characterized by night blindness and progressive loss of visual field due to degeneration of photoreceptors. RP has been reported to affect not only the retina but also the ocular dimensions [2, 3]. The alterations in ocular dimensions in patients with RP could be associated with a high prevalence of keratoconus [4], angle-closure glaucoma [5], and certain refractive errors, such as myopia and astigmatism [6]. Although there are a few reports that describe the alterations in the anterior segment [2, 3], detailed ocular biometry in patients with RP is not yet fully understood.

The IOLMaster 700 (Carl Zeiss AG, Oberkochen, Germany) is the first swept-source optical coherence tomography (OCT)-based biometry device that provides anatomical details of the complete longitudinal section of the eye; all axial measurements including axial length, central corneal thickness (CCT), anterior chamber depth (ACD), and lens thickness can be obtained. Additionally, it combines telecentric keratometry and OCT technology to measure both anterior and posterior corneal curvatures.

Recently, accumulated knowledge of genetic findings in RP has shown various clinical features among different causative genes. Some causative genes have specific pathological conditions complicated with the retinal dystrophy, for example, high myopia in *RPGR* variants [7] or angle-closure glaucoma in *CRB1* and *MFRP* variants [8–10]. These reports have suggested that patients with RP may also have different alterations in ocular dimensions among different causative genes. In the Japanese population, the most prevalent causative gene is *EYS*, which accounts for 30% of genetically solved cases [11–14]. Thus, it is the most promising causative gene for which clinical data are being accumulated in Japan. To the best of our knowledge, this is the first study to describe the ocular dimension features of patients with *EYS*-related RP. This pilot study aimed to investigate the hypothesis that different causative genes are associated with different ocular biometry findings, by identifying the features of IOLMaster 700-based total ocular biometry in patients with *EYS*-related RP.

## Materials and methods

This retrospective study included patients diagnosed with RP who were followed up at the Kobe City Eye Hospital between June 2020 and December 2020. RP was diagnosed according to the guidelines of the Japanese Ophthalmological Society [15], based on clinical history, fundus appearance, visual fields, and full field electroretinogram results. Cataract was defined as the presence of nuclear (greater than grade 1 of Emery-Little classification), cortical, or posterior subcapsular (PSC) cataract. The data related to the presence or absence of cataract were retrieved from the medical records as recorded by one or more of the three ophthalmologists who were in charge of the inherited retinal disease clinic of our hospital. The patients were included in the study only if the medical records had ocular biometry measurements with the IOLMaster 700 and genetic diagnostic tests. Patients with a history of ocular surgery, including cataract extraction, corneal or other anterior segment diseases that could lead to corneal opacification, or astigmatism were excluded from the study. Among the 44 patients who met these criteria, genetic diagnostic tests identified *EYS* variants in 18, *USH2A* variants in 4, and other gene variants in 11 patients. Eleven cases remained genetically unsolved with our multi gene-panel analysis as described in the later section “Genetic analysis”. The genetic diagnoses of the patients are shown in Table 1. Following this distribution of causative genes, we planned to use the clinical data of patients with variants other than *EYS* as the control group. None of the patients had causative genes that could affect ocular biometry, such as *RPGR*, *CRB1*, and *MFRP.* We divided the patients into two groups: *EYS*-related RP (35 eyes of 18 patients with *EYS* variants) and other RP (51 eyes of 26 patients with other variants and genetically unsolved cases). Genetic variants in the patients with *EYS*-related RP included in this study are presented in Additional file 1.

**Table 1:** Genetic diagnosis of patients with retinitis pigmentosa

Genetic variants	Number of patients
EYS	18
USH2A	4
PDE6B	1
RPE65	1
C2orf71	1
MAK	1
PRPF31	1
PROM1	1
NHPH1	1
RHO	1
NRL	1
IMPDH	1
TOPORS	1
Genetically unsolved	11

### Ocular biometry using the IOLMaster 700

Biometric measurements were performed before pupillary dilatation in all patients. The records of all measurements, including axial length, keratometry, CCT, ACD, aqueous depth, lens thickness, and corneal diameter (white to white), were extracted. ACD is the distance between the tear film and the anterior lens capsule, and aqueous depth is the distance between the corneal endothelium and anterior lens capsule. Total keratometry is a new measurement that combines telecentric keratometry and OCT technology. In addition to standard keratometry in which the corneal curvature radius at the flattest meridian (R1) and steepest meridian (R2) is measured, posterior corneal surface measurements (PR1 and PR2) and total keratometry reading (TR1 and TR2) can be obtained.

### Corneal astigmatism measurements

We analysed three corneal astigmatism values based on keratometry measurements: (1) standard keratometric astigmatism (KA), (2) posterior corneal astigmatism (PCA), and (3) total corneal astigmatism (TCA) [16]. The following formulae were used:

(1) Standard KA (1.3375–1) × 1000 / R2—(1.3375–1) × 1000 / R1.

The keratometric refractive index of 1.3375 was used to convert R into corneal power. (2) PCA (1.336–1.376) × 1000 / PR2—(1.336–1.376) × 1000 / PR1. The refractive indices of the cornea and aqueous humour were 1.336 and 1.376, respectively, and were used to convert PR into corneal power. (3) TCA. (1.3375–1) × 1000 / TR2—(1.3375–1) × 1000 / TR1 The keratometric refractive index of 1.3375 was used to convert TR into corneal power

### Genetic analysis

Genetic testing was performed by stepwise Sanger-sequencing using one or two panels of 15 and 27 genes in 2008–2015 [13] or next-generation sequencing using panels of 39 or 50 genes in 2015–2019 [17] or 2019 and later [18], respectively. The patients in this study underwent one of these screenings for genetic diagnosis tests.

### Statistical analysis

All statistical analyses were performed using SPSS for Windows, version 25 (SPSS Inc., Chicago, IL, USA). The demographics and biometric parameters of *EYS*-related RP and other RP were compared. The normality of the data distribution was tested using the Kolmogorov–Smirnov test; age and WTW were non-normally distributed. Differences in age were compared using the Mann–Whitney U test. The ratio of female to male patients was compared using the chi-square test. A generalized estimating equation model was used for other variables to account for the correlation between both eyes of a single patient. Statistical significance was set at *P* < 0.05.

## Results

A total of 86 eyes of 44 patients (21 males and 23 females) with RP were included. The demographic data and ocular biometric parameters of the patients are summarised in Table 2. The mean median age (interquartile range) was 51.0 (20.0) years (range: 21–68 years). There were 51 eyes with clear lenses and 45 eyes with cataracts (including 13 eyes with PSC). Eyes with intraocular lenses were excluded from the study.

**Table 2:** Patient demographics and ocular biometric parameters

	**Overall**	***EYS*-related RP**	**Other RP**	*P* value^*^
	44 patients	18 patients	26 patients	
**Age (years), median [IQR]**	51.0 [20.0]	49.5 [18.0]	51.0 [29.0]	0.748
**Female, n (%)**	23 (52.3)	12 (66.7)	11 (42.3)	0.112
	86 eyes	35 eyes	51 eyes	
**Cataract, n (%)**	45 (52.3)	19 (54.3)	26 (51.0)	0.829
**PSC, n (%)**	13 (15.1)	10 (28.6)	3 (5.9)	0.045*
**Biometric parameters**^**a**^				
**AL (mm)**	24.2 (23.9–24.5)	24.3 (23.8–24.8)	24.1 (23.9–24.4)	0.610
**CCT (μm)**	526.0 (520.6–531.4)	515.4 (506.9–523.9)	536.6 (529.8–543.4)	< 0.001*
**ACD (mm)**	3.05 (2.98–3.13)	3.05 (2.92–3.17)	3.06 (2.98–3.14)	0.861
**AQD (mm)**	2.53 (2.45–2.60)	2.53 (2.40–2.66)	2.52 (2.44–2.61)	0.922
**LT (mm)**	4.48 (4.39–4.57)	4.53 (4.38–4.67)	4.44 (4.32–4.55)	0.357
**WTW (mm)**	12.1 (12.0–12.1)	12.1 (12.0–12.2)	12.0 (11.9–12.1)	0.349
**Keratometry**				
**R1 (mm)**	7.84 (7.79–7.89)	7.82 (7.74–7.89)	7.87 (7.80–7.93)	0.316
**R2 (mm)**	7.56 (7.51–7.61)	7.52 (7.43–7.60)	7.61 (7.56–7.66)	0.072
**PR1 (mm)**	6.99 (6.94–7.04)	6.96 (6.88–7.04)	7.02 (6.96–7.08)	0.208
**PR2 (mm)**	6.61 (6.56–6.67)	6.55 (6.45–6.65)	6.68 (6.63–6.73)	0.024*
**TR1 (mm)**	7.83 (7.78–7.88)	7.81 (7.74–7.88)	7.86 (7.80–7.92)	0.331
**TR2 (mm)**	7.57 (7.52–7.62)	7.52 (7.44–7.61)	7.61 (7.55–7.67)	0.102
**Astigmatism**				
**Standard KA (D)**	1.59 (1.40–1.78)	1.75 (1.44–2.06)	1.43 (1.22–1.64)	0.093
**PCA (D)**	-0.33 (-0.37 to -0.29)	-0.37 (-0.42 to -0.31)	-0.29 (-0.34 to -0.25)	0.061
**TCA (D)**	1.52 (1.33–1.70)	1.65 (1.35–1.96)	1.38 (1.18–1.58)	0.145

Abbreviations: *RP*, retinitis pigmentosa, *IQR*, interquartile range, *PSC*, posterior subcapsular cataract, *AL*, axial length, *CCT*, central corneal thickness, *ACD*, anterior chamber depth, *AQD*, aqueous depth, *LT*, lens thickness, *WTW*, white to white, *R1* and *R2*, standard keratometry corneal curvature radius at the flattest and steepest meridians, respectively, *PR1* and *PR2*, posterior corneal curvatures radius at the flattest and steepest meridian, respectively, *TR1* and *TR2*, total keratometry corneal curvature radius at the flattest and steepest meridians respectively *KA*, keratometric astigmatism *PCA*. posterior corneal astigmatism *TCA*, total corneal astigmatism

^*^Statistically significant at the *P* < 0.05 level (generalized estimating equation model)

^a^Eye-level values are represented as the estimated marginal mean (95% confidence interval)

A comparison between patients with *EYS*-related RP and those with other RP is also shown in Table 2. There was no significant difference in sex and the presence of cataract between the two groups. The frequency of PSC was significantly higher in patients with *EYS*-related RP (28.6%) than in those with other RP (5.9%) (*P* = 0.045). CCT was significantly thinner in patients with *EYS*-related RP than in those with other RP (*P* < 0.001). In posterior keratometry, corneal curvature radius at the steepest meridian was significantly smaller in patients with *EYS-*related RP (*P* = 0.024). In standard and total keratometry, corneal curvature radius at the steepest meridian (R2 and TR2) was smaller in patients with *EYS-*related RP, although these differences were not statistically significant. The magnitudes of all corneal astigmatism measurements, namely standard KA, PCA, and TCA, were greater in patients with *EYS*-related RP, although these differences were not statistically significant.

## Discussion

This study presents the total ocular biometry measurements in Japanese patients with RP. We observed some unique features of ocular dimensions in patients with *EYS*-related RP. First, CCT was thinner in patients with *EYS*-related RP than in those with other RP. Second, corneal curvature radius at the steepest meridian was smaller in patients with *EYS*-related RP than in those with other RP. Küçük et al. have reported that Turkish patients with RP had thinner CCT (mean: 518 mm), higher maximum keratometry values [mean: 45.85 D (equivalent to a corneal curvature radius of 7.36 mm)], and smaller anterior chamber angle on Scheimpflug imaging compared to those of healthy controls [mean CCT: 534 mm, mean maximum keratometry: 44.69 D (equivalent to a corneal curvature radius of 7.55 mm)]. They suggested that patients with RP may have different anterior segment parameters compared with healthy subjects; however, genetic information was not presented [3]. In our study, the patients with *EYS*-associated RP had thinner CCT and higher maximum keratometry values, as also reported by Küçük et al., than the patients with other RP. Our results suggest that these unique features in patients with RP are associated with certain causative genes, such as *EYS*. Furthermore, the above-mentioned corneal alterations could be associated with astigmatism. A previous study on corneal astigmatism measurement using the IOLMaster 700 in the Chinese population reported a mean standard KA of 1.00 D, mean PCA of -0.19 D, and mean TCA of 1.03 D [16], all of which are smaller in magnitude compared to those of our patients (mean standard KA: 1.59 D, mean PCA: -0.33 D, and mean TCA: 1.52 D). The greater magnitude of corneal astigmatism may contribute to the reported high prevalence of astigmatism in patients with RP [6], especially in patients with *EYS*-related RP, as observed in our cohort.

The mechanism of corneal alteration in patients with RP has not yet been explained. RP has been reported as a risk factor for keratoconus, which is characterized by progressive thinning of the cornea [19]. Low-grade inflammation during the course of retinal degeneration in RP is considered the pathological mechanism underlying keratoconus. PSC is the most common subtype of RP-related cataracts, and inflammation also plays a major role in the formation of PSC in RP [20]. In our cohort, 15.1% of patients had PSC, and its prevalence was higher in patients with *EYS*-related RP (28.6%) than in those with other RP (5.9%), suggesting that the severity of inflammation may contribute to different degrees of corneal alteration in RP.

*EYS* variants were first reported to have a pathogenic effect on RP in 2008 [21, 22]. It has four isoforms all of which are mainly expressed in the human retina; however, its expression in the cornea has not yet been reported. The *EYS* protein is considered to play a role in maintaining the stability of the ciliary axoneme in photoreceptors [23]. To date, there are no findings that explain the association between the *EYS* variant and corneal alterations. Future studies are needed to understand the underlying mechanisms of our results.

To the best of our knowledge, this is the first report to describe the ocular dimension features of patients with *EYS*-related RP. A major limitation of this study was that each of the genetic variants other than *EYS* had a small sample size, which limits our ability to understand their respective characteristics. Another limitation was the retrospective nature of the study design. Additionally, statistical analysis of the comparison between RP eyes and normal eyes was lacking because we did not have a control group of normal subjects in this study. Finally, we used different panels for genetic testing. Using older methods might contribute to detection failure of some causative genes; however, *EYS* was included in all the panels we used. Further studies including a large number of patients and a control group are required to validate the unique findings of ocular biometry in patients with RP.

In conclusion, patients with *EYS*-related RP had some unique features of ocular dimensions, such as thinner CCT and smaller posterior corneal curvature radius at the steep meridian compared to patients with other RP. The preliminary results of this study suggest that patients with RP have different ocular dimension features among the different causative genes.

## Supplementary Information

**Additional file 1: Supplementary Table 1.** Description of data: Genetic variants in the patients with EYS-related retinitis pigmentosa.

## Data Availability

All data included in this study are available from the corresponding author upon request.

## Abbreviations

ACD: Anterior chamber depth
AQD: Aqueous depth
CCT: Central corneal thickness
KA: Keratometric astigmatism
LT: Lens thickness
OCT: Optical coherence tomography
PCA: Posterior corneal astigmatism
PR1: Posterior corneal curvature radius at the flattest meridian
PR2: Posterior corneal curvature radius at the steepest meridian
PSC: Posterior subcapsular cataract
RP: Retinitis pigmentosa
R1: Corneal curvature radius at the flattest meridian
R2: Corneal curvature radius at the steepest meridian
TCA: Total corneal astigmatism
TR1: Total keratometry corneal curvature radius at the flattest meridian
TR2: Total keratometry corneal curvature radius at the steepest meridian
WTW: White to white

## References

[1] Hartong DT, Berson EL, Dryja TP (2006). Retinitis pigmentosa. The Lancet.

[2] Horneber M, Gottanka J, Milam AH, Lütjen-Drecoll E (1996). Alterations in anterior segment dimensions in eyes with retinitis pigmentosa. Graefes Arch Clin Exp Ophthalmol.

[3] Küçük B, Yıldırım Y, Özsaygılı C (2019). Anterior chamber characteristics assessed by rotating Scheimpflug imaging in patients with retinitis pigmentosa. Arq Bras Oftalmol.

[4] Grünauer-Kloevekorn C, Duncker GI (2006). Keratoconus: epidemiology, risk factors and diagnosis. Klin Monbl Augenheilkd.

[5] Ko YC, Liu CJ, Hwang DK, Chen TJ, Liu CJ (2014). Increased risk of acute angle closure in retinitis pigmentosa: a population-based case-control study. PLoS ONE.

[6] Sieving PA, Fishman GA (1978). Refractive errors of retinitis pigmentosa patients. Br J Ophthalmol.

[7] Jin ZB, Liu XQ, Hayakawa M, Murakami A, Nao-I N (2006). Mutational analysis of RPGR and RP2 genes in Japanese patients with retinitis pigmentosa: identification of four mutations. Mol Vis.

[8] Carricondo PC, Andrade T, Prasov L, Ayres BM, Moroi SE. Nanophthalmos: a review of the clinical spectrum and genetics. J Ophthalmol. 2018;2018:2735465. 10.1155/2018/2735465. 10.1155/2018/2735465 PMC5971257 29862063

[9] Ayala-Ramirez R, Graue-Wiechers F, Robredo V, Amato-Almanza M, Horta-Diez I, Zenteno JC (2006). A new autosomal recessive syndrome consisting of posterior microphthalmos, retinitis pigmentosa, foveoschisis, and optic disc drusen is caused by a MFRP gene mutation. Mol Vis.

[10] Crespí J, Buil JA, Bassaganyas F, Vela-Segarra JI, Díaz-Cascajosa J, Ayala-Ramirez R (2008). A novel mutation confirms MFRP as the gene causing the syndrome of nanophthalmos-renititis pigmentosa-foveoschisis-optic disk drusen. Am J Ophthalmol.

[11] Iwanami M, Oshikawa M, Nishida T, Nakadomari S, Kato S (2012). High prevalence of mutations in the *EYS* gene in Japanese patients with autosomal recessive retinitis pigmentosa. Invest Ophthalmol Vis Sci.

[12] Oishi M, Oishi A, Gotoh N, Ogino K, Higasa K, Iida K (2014). Comprehensive molecular diagnosis of a large cohort of Japanese retinitis pigmentosa and Usher syndrome patients by next-generation sequencing. Invest Ophthalmol Vis Sci.

[13] Arai Y, Maeda A, Hirami Y, Ishigami C, Kosugi S, Mandai M (2015). Retinitis pigmentosa with *EYS* mutations is the most prevalent inherited retinal dystrophy in Japanese populations. J Ophthalmol.

[14] Koyanagi Y, Akiyama M, Nishiguchi MK, Momozawa Y, Kamatani Y, Takata S (2019). Genetic characteristics of retinitis pigmentosa in 1204 Japanese patients. J Med Genet.

[15] Yamamoto S, Murakami A, Takahashi M, Ikeda Y, Hirami Y, Nakamura Y (2016). Clinical practice guidelines for retinitis pigmentosa. Nippon Ganka Gakkai Zasshi.

[16] Wang L, Cao D, Vilar C, Koch DD (2020). Posterior and total corneal astigmatism measured with optical coherence tomography-based biometer and dual Scheimpflug analyzer. J Cataract Refract Surg.

[17] Maeda A, Yoshida A, Kawai K, Arai Y, Akiba R, Inaba A (2018). Development of a molecular diagnostic test for retinitis pigmentosa in the Japanese population. Jpn J Ophthalmol.

[18] Inaba A, Maeda A, Yoshida A, Kawai K, Hirami Y, Kurimoto Y (2020). Truncating variants contribute to hearing loss and severe retinopathy in USH2A-associated retinitis pigmentosa in Japanese patients. Int J Mol Sci.

[19] Bawazeer AM, Hodge WG, Lorimer B (2000). Atopy and keratoconus: a multivariate analysis. Br J Ophthalmol.

[20] Fujiwara K, Ikeda Y, Murakami Y, Funatsu J, Nakatake S, Tachibana T (2017). Risk factors for posterior subcapsular cataract in retinitis pigmentosa. Invest Ophthalmol Vis Sci.

[21] Abd El-Aziz MM, Barragan I, O’Driscoll CA, Goodstadt L, Prigmore E, Borrego S (2008). EYS, encoding an ortholog of Drosophila spacemaker, is mutated in autosomal recessive retinitis pigmentosa. Nat Genet.

[22] Collin RWJ, Littink KW, Klevering BJ, van den Born LI, Koenekoop RK, Zonneveld MN (2008). Identification of a 2 Mb human ortholog of Drosophila eyes shut/spacemaker that is mutated in patients with retinitis pigmentosa. Am J Hum Genet.

[23] Alfano G, Kruczek PM, Shah AZ, Kramarz B, Jeffery G, Zelhof AC (2016). EYS is a protein associated with the ciliary axoneme in rods and cones. PLoS One.

