# Supplementary material for: Ocular biometry with swept-source optical coherence tomography-based optical biometer in Japanese patients with EYS-related retinitis pigmentosa: a retrospective study
Source: BMC Ophthalmol. 2022 Feb 2;22:51. doi: 10.1186/s12886-022-02284-3 (PMC8811986; doi:10.1186/s12886-022-02284-3)
Supplement: Supplementary file 1 — Additional file 1: Supplementary Table 1. Description of data: Genetic variants in the patients with EYS-related retinitis pigmentosa. [file 12886_2022_2284_MOESM1_ESM.pdf]

Supplementary Table 1. Genetic variants in the patients with *EYS*-related retinitis pigmentosa.

Subject number	Age	Sex	Mutation	
EYS-1	36	Male	c.8805C>G	c.4957dupA
EYS-2	51	Male	c.8805C>G	c.2528G>A
EYS-3	24	Male	c.8805C>A	c.5794G>T
EYS-4	57	Male	c.1628_1631delGTGA	c.4957dupA
EYS-5	40	Female	c.4957dupA	c.6557G>A
EYS-6	58	Male	c.1299+1G>T	c.8805C>G
EYS-7	42	Female	c.4957dupA	c.6557G>A
EYS-8	54	Female	c.8805C>G	c.4957dupA
EYS-9	57	Female	c.8805C>A	c.6557G>A
EYS-10	66	Female	c.4957dupA	c.2528G>A
EYS-11	37	Female	c.4957dupA	c.4957dupA
EYS-12	48	Female	c.4957dupA	c.8868C>A
EYS-13	61	Female	c.8196_8200delCTTTC	c.8196_8200delCTTTC
EYS-14	39	Female	c.4957dupA	c.4957dupA
EYS-15	40	Female	c.6541delA	ex32-33dup
EYS-16	47	Female	c.4957dupA	c.2528G>A
EYS-17	62	Female	c.8805C>G	ex6-8del
EYS-18	57	Male	c.2528G>A	c.2528G>A
